# Impact of HIV-associated cognitive impairment on functional independence, frailty and quality of life in the modern era: a meta-analysis

**DOI:** 10.1038/s41598-022-10474-8

**Published:** 2022-04-19

**Authors:** Martins Nweke, Nombeko Mshunqane, Nalini Govender, Aderonke O. Akinpelu, Maryjane Ukwuoma

**Affiliations:** 1 Department of Physiotherapy, Faculty of Health Sciences, University of Pretoria, Pretoria, 0028 Hatfield South Africa; 2 Department of Basic Medical Sciences, Durban University of Technology, Durban, South Africa; 3 Department of Physiotherapy, University of Ibadan, Ibadan, Nigeria; 4 Department of Physiotherapy, University of Nigeria Teaching Hospital Ituku-Ozalla, Enugu, Nigeria

**Keywords:** Physiology, Health care

## Abstract

HIV-associated neurocognitive disorder (HAND) is an important sequela of HIV infection. Combined antiretroviral therapy (cART) has improved the health outcomes of many people living with HIV but has given rise to a less severe but limiting form of HAND. The study aimed to evaluate the impact of HAND on medication adherence, activities of daily living (ADL), quality of life and frailty. This systematic review adheres to the guidelines for Preferred Reporting Items for Systematic Reviews and Meta-Analyses. We searched MEDLINE, PubMed, CINAHL, Academic Search Complete, and PsycINFO online databases. Studies were included if they examined the relationship between HAND and medication adherence, ADL, quality of life and frailty, and were conducted between 1997 and 2021. We used a random-effects meta-analysis model to assess the impact of HAND on outcome variables. Forty papers, totaling 11,540 participants, were included in the narrative and quantitative syntheses. Cognitive impairment was associated with poorer medication adherence (r = 0.601, CI 0.338 to 0.776, *p* = 0.001, I^2^ = 94.66). Cognitive impairment did not influence ADL (r = 0.167, CI-0.215 to 0.505, *p* = 0.393) and quality of life (r = 0.244, CI 0.117 to 0.548, *p* = 0.182). In the cART era, HAND appears to be associated with adherence to medication, which may influence future health outcomes. In PLWHIV who are adherent to cART, cognitive impairment does not appear to interfere with ADL and quality of life.

## Introduction

Human immunodeficiency virus (HIV) infection has evolved into a chronic disease, which has led to the manifestation of HIV-associated neurocognitive disorder (HAND)^1^ impacting medication adherence and quality of life (QOL)^2,3^. Before combination antiretroviral therapy (cART) was widely used, severe forms of HAND were more prevalent, with HIV-associated dementia (HAD) affecting almost 50% of PLWHIV^4^. For PLWHIV, HAD was a strong predictor of death, functional dependence, geriatric-like frailty and falls, and poor QOL^4,5^. Since the introduction of cART, HAND has become a less common but persistent problem^6^. Currently, the global prevalence of HAND is 23.5%, with Latin America and the Caribbean possessing the greatest burden (50.6%), followed by western and central Europe and North America (24.3%), sub-Saharan Africa (19.0%) AND Asia (18.3%)^7^. Similarly, the burden of HAND varies with economic strength and lifestyle, with low, middle and high-income countries possessing prevalence estimates of 11.4%, 26.6% and 23.3%^7^. Typically, HAND encompasses a wide spectrum of cognitive-behavioural and motor deficits^8,9^. Over the years, the manifestation of HAND has changed, with milder forms of neurocognitive impairment becoming more common. Clinical images have revealed that the regions of the brain affected by HAND have also changed in the cART era^10,11^. Before cART, subcortical damage afflicting the basal ganglia was associated with functional impairment, which has changed to more subtle and insidious cortical damage mainly in the hippocampus and the temporal cortex leading to greater deficits in executive functioning and working memory^12–14^. Extrapyramidal signs are now less common in patients with HAND, who are on cART^14^. Before cART, PLWHIV showed an increase in age-dependent glial activation and neuronal damage leading to accelerated ageing, while in the modern era, cART-treated individuals show only signs of premature ageing^15^. It is not clear whether changing clinical HAND phenotypes have impacted outcomes such as medication adherence, employment and QOL of PLWHIV. Current evidence suggests that HAND has become less debilitating, but still has the potential to reduce medication adherence, QOL, limiting activities of daily living (ADL), leading to functional dependence^16–18^. Existing literature presents conflicting evidence. While some studies report that HAND has a strong influence on medication adherence^19^, ADL (16), and QOL^17^, other studies have reported weak or no association between HAND and medication adherence^18^, ADL^20,21^, frailty^22^, and QOL^23^. Two systematic reviews^3,24^, have tried to synthesize existing evidence but did not conduct a meta-analysis of findings. A significant number of publications have recently emerged, necessitating a meta-analysis of current evidence.

Given that HAND seems common patients on cART worldwide^7^, it is essential to quantify and cumulatively establish its functional consequences of HAND vis-a-vis medication adherence, ADL, frailty and QOL. By quantifying the functional consequences of HAND in the cART era, we will be providing relevant stakeholders with up to date information that will inform current strategies and treatment guidelines for HAND. The study aimed to evaluate the impact of HAND on medication adherence, ADL, frailty and QOL in the modern era.

## Methods

### Protocol and registration

This systematic review adheres to the guidelines for Preferred Reporting Items for Systematic Reviews and Meta-Analyses (PRISMA)^25^. The protocol is registered with PROSPERO─ CRD42021240726.

### Eligibility criteria

This is a systematic review of observational studies reporting the relationship of HAND with medication adherence, activities of daily living, frailty, and quality of life. This review included peer-reviewed literature written in the English language, irrespective of location, sample size and test statistics. We included studies in which trained persons using a measure of domain or global cognitive function/impairment assessed cognitive function/impairment. We included studies that assessed any or all the primary outcomes namely medication adherence, activities of daily living, quality of life and frailty. Psychosocial variables and sociodemographic and study characteristics constituted the secondary outcomes.

### Inclusion criteria

- Peer-reviewed articles report the relationship of HAND with any or all medication adherence, activities of daily living/work, frailty, and quality of life.
- Studies conducted in which the assessment tool and/or assessor's qualification was stated
- Peer review articles published after 1996 (pre-cART era).

### Exclusion criteria

- Studies reporting medication adherence, activities of daily living, frailty, and/or Quality of life in PLWHIV but in which their relationship with HAND was not explored and reported.
- Peer-reviewed articles reporting the relationship of HAND with any or all medication adherence, activities of daily living, frailty, and quality of life but in which the assessment tool and/or assessor's qualification was not stated.
- Peer-reviewed articles reporting the relationship of HAND with any or all medication adherence, activities of daily living, frailty, and quality of life but published on/before 1996.

### Information source

We searched five online bibliographic databases namely MEDLINE, PubMed, CINAHL, Academic Search Complete, and PsycINFO using medical subject headings (MeSH), and keywords identified in the title, abstract and/or text of articles. The last search was conducted in February 2021, with data collection spanning between February and August 2021.

### Search

The search strategy was piloted in PubMed. After trying several combinations of terms, the most sensitive strategy was chosen and reported. Sensitivity was judged at face value. We adapted the strategy to the syntax and subject headings of the remaining databases (Appendix 1).

### Study selection

**Figure 1:**
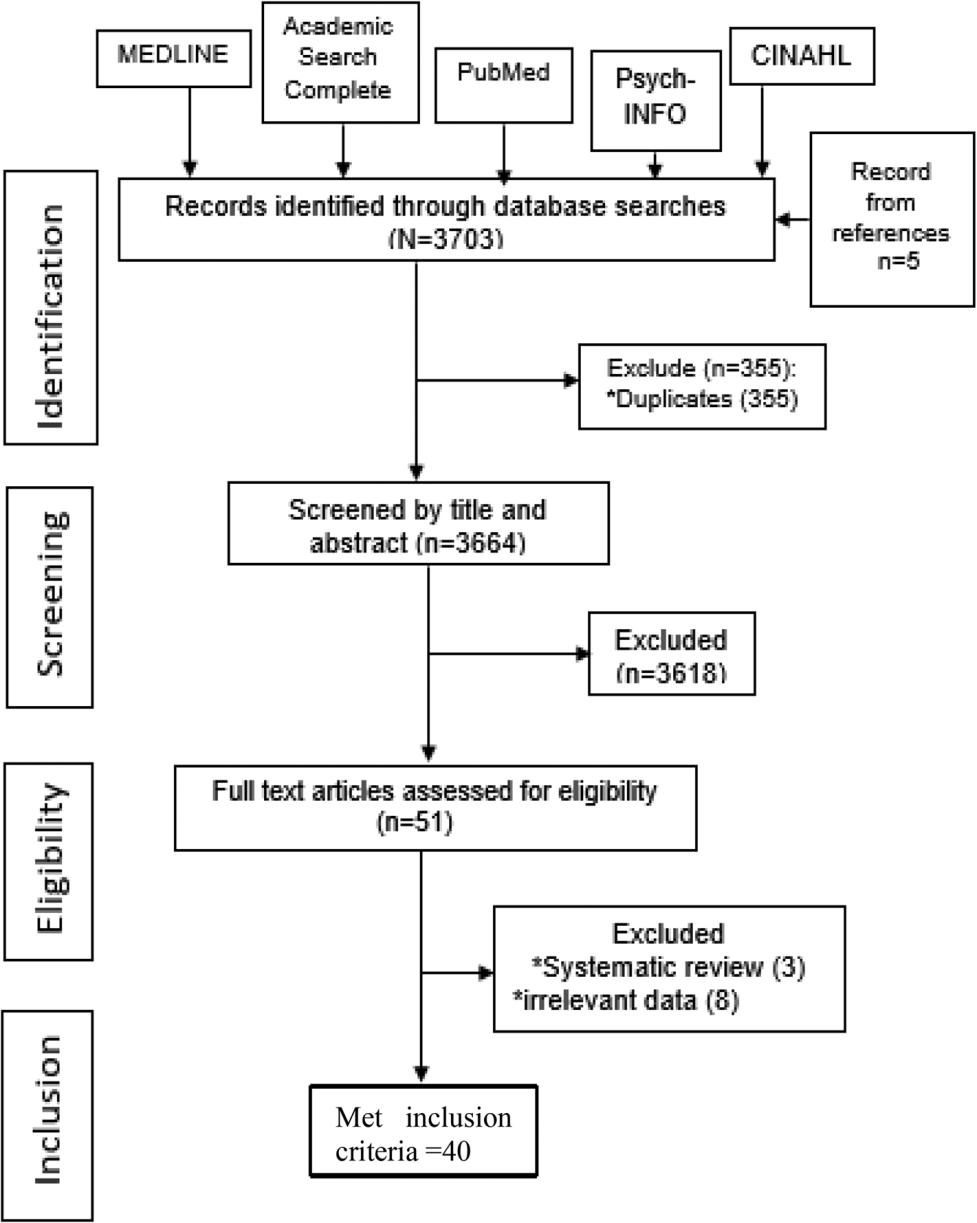
PRISMA flow diagram of the systematic review of articles estimating the impact of HAND on medication adherence, activitiesof daily living quality of life and frailty in the modern era (1997–2021).

We exported our search results directly into EndNote 8, where duplicate articles were removed. Once all the duplicate articles were removed, the primary reviewer screened the titles and selected articles that met the inclusion criteria. Two reviewers independently reviewed the full-text articles and excluded articles that did not meet the eligibility criteria. We screened the reference lists of relevant articles to identify additional studies. We did not restrict studies based on target population, setting, neuropsychological test used or language. We only included studies published in or after 1997. We only included studies published in English. We excluded case reports to enhance comparability. We excluded studies conducted on high-risk groups such as individuals with traumatic brain injury. We conducted a meta-analysis for outcomes, provided there were at least three studies reporting summary estimates for the outcome. The PRISMA diagram details the flow of studies throughout the selection process, along with reasons for excluding articles (Fig. 1).

### Data collection process

In this review, we undertook independent data extraction. From each article, we extracted primary data including summary estimates (odds ratio (OR) or correlation coefficient (r)) describing the relationship between cognitive functioning/impairment and medication adherence, ADL, frailty and QOL, among PLWHIV. We also extracted secondary data, namely age, gender and education from each article. We also recorded article information including author, title, population, sample size, sampling techniques, diagnostic criteria/method of assessment, country and summary of findings. Data were extracted using a custom spreadsheet. We did not have to contact any authors because we were able to retrieve the full texts of all the articles. We excluded five full texts, three systematic reviews and two studies with non-relevant data.

### Data items

Primary data sought included medication adherence, the activity of daily living, frailty and quality of life. Secondary data were age, gender, educational qualification. Other information gathered included article were authors' name, study title, study population, study sample size, sampling techniques, and diagnostic criteria/method of assessment, country and summary of finding.

### Risk of bias assessment

We assessed the quality of the included studies to validate our findings and improve the value of the study for stakeholders and consumers of health information. This was necessary as stakeholders and consumers of health information should exercise the best judgment when determining the economic importance of disorders like HAND, and a treatment path. The risk of bias assessment was carried out independently and with the aid of the mixed method appraisal tool (MMAT) Version 2011^26^. The risk was classified as low for a quality score 80–100%, medium for a quality score 40–60 and high for a score less than 40.

### Summary measures

The impact of HAND on medical adherence, ADL, frailty and QOL were estimated using correlation coefficient. We converted odds ratios and Pearson chi-square statistics into correlation coefficients following the approach used in Borenstein et al.^27^ and Digby in Bonnet et al.^28^. We preferred Borenstein & colleagues for *p* > 0.05 and Digby^29^ for *p* < 0.05. Pearson Chi-square statistics were converted to correlation coefficients using Wilson's effect size calculator^30^. We used the McGrath calculator to calculate the mean and standard deviation for three studies that summarized age using median and inter-quartile ranges^31^. For quantitative synthesis, we grouped sleep, employment and driving as ADL. One study reported the relationship between cognitive function and medication management, which we grouped under medication adherence.

### Synthesis of results

In this review, measures of heterogeneity and study outcomes were sorted by the author and presented in an evidence table (Appendix 2). We used a random-effects model of meta-analysis to estimate the association of HAND with functional outcomes. We conducted the meta-analysis using MedCalc, with α set at 0.05.

### Risk of bias across studies and additional analysis

We evaluated the risk of bias across studies by examining the publication bias, which was assessed using Egger's test. We conducted a sensitivity analysis for each outcome, including only studies examining the relationship between global cognitive impairment and the pre-specified outcome.

### Strength of evidence

We assessed the strength of evidence using the grading of recommendations assessment, development and evaluation (GRADE)^7^. We assessed four domains of the GRADE namely risk of bias, consistency directness, and precision. The risk of bias was classified into low, medium and high as previously described. We assessed consistency based on the sign of the effect estimates obtained in the individual studies. We assessed whether a proxy outcome was used in individual studies. Precision was based on the width of the confidence interval^32–34^.

## Results

### Study selection and characteristics

Our initial search returned 3698 articles. After removing duplicates, 3664 articles remained. After screening all the titles and abstracts, we excluded 3613 irrelevant records, leaving 51 articles for full-text review. Of the 51 full-texts, we excluded 11 articles. Ultimately, we reviewed 40 articles comprising 11,540 participants from six countries (Fig. 1, Appendix 2). The sample sizes varied from 37^35^ to 1306^23^. More than 80% of the studies were conducted in the United States, with 9808 participants or 85% of the total sample population. Two studies were conducted in Italy and one study each from Canada, South Africa, India, Malaysia and Kenya. Thirty-three (82.5%) studies used non-probability sampling methods while six (15%) studies used a random sampling strategy. Regarding study design, 23 (57.5%) and 16 (40%) were longitudinal and cross-sectional, respectively (Appendix 2).

### Strength of evidence and result of individuals studies

The cumulative confidence in this review seems as revealed by the strength of evidence was high as the included studies possessed low to medium risk of bias. Specifically, 4 studies possessed a moderate risk of bias, while 36 possessed a low risk of bias. Generally, evidence was consistent, direct and precise except for the evidence on the impact of HAND on activities of daily living which were inconsistent and imprecise (Table 1).

**Table 1:** Strength of evidence.

	Risk of bias (limitation)	Is sampling strategy relevant?	Is the sample representative of the target population?	Are the measurements appropriate?	Is the risk of nonresponse bias low?	Does statistical analysis appropriate?	Total score	The overall risk of bias
1	Avants et al. 2001	Yes	Yes	Yes	Yes	Yes	5	low
	Andrade et al. 2013	Yes	Not clear	Yes	Not clear	Yes	3	Medium
	Barclay et al. 2007	Yes	Yes	Yes	Not clear	Yes	4	Low
	Becker et al. 2011	Yes	Yes	Yes	Not clear	Yes	4	Medium
	Byun et al. 2016	Yes	Not clear	Yes	Not clear	Yes	3	Medium
	Caballero et al. 2018	No	No	Yes	Yes	Yes	3	Medium
	Chernoff et al. 2010	Yes	Yes	Yes	Yes	Yes	5	Low
	Cook et al. 2016	Yes	Yes	Yes	Yes	Yes	5	Low
	Doyle et al. 2012	Yes	Not clear	Yes	Yes	Yes	4	Low
	Erlandson et al. 2018	Yes	Yes	Yes	Yes	Yes	5	Low
	Ettenhofer et al. 2009	Yes	Yes	Yes	Yes	Yes	5	Low
	Ettenhofer et al. 2010	Yes	Not clear	Yes	Yes	Yes	4	Low
	Gouse et al. 2020	Yes	Not clear	Yes	Yes	Yes	4	Low
	Harrison et al. 2017	Yes	Yes	Not clear	Yes	Yes	4	Low
	Hinkin et al. 2004	Yes	Not clear	Yes	Yes	Yes	4	Low
	Hinkin et al. 2002	Yes	Not clear	Yes	Yes	Yes	4	Low
	Jones et al. 2019	Yes	Yes	Yes	Yes	Yes	5	Low
	Marquine et al. 2018	Yes	Yes	Yes	Yes	Yes	5	Low
	Masters et al. 2021	Yes	Yes	Yes	Not clear	Yes	4	Low
	Mayo et al. 2020	Yes	Yes	Yes	Not clear	Not clear	3	Medium
	Moore et al. 2014	Yes	Yes	Yes	Yes	Yes	5	Low
	Nyongesa et al. 2018	Yes	Yes	Yes	Yes	Yes	5	Low
	Oppenheim et al. 2018	Yes	Yes	Yes	Yes	Yes	5	Low
	Schifitto et al. 2001	Yes	Not clear	Yes	Not clear	Yes	3	Medium
	Shrestha et al. 2017	Yes	Yes	Yes	Yes	Yes	5	Low
	Smith 2012	Yes	Yes	Yes	Yes	Not clear	4	Low
	Solomon and Halkitis (2008)	Yes	Yes	Yes	Yes	Yes	5	Low
	Thaler et al. 2015	Yes	Yes	Yes	Yes	Yes	5	Low
	Thames et al. 2011	Yes	Yes	Yes	Yes	Yes	5	Low
	Tierney et al. 2019	Yes	Yes	Yes	Yes	Yes	5	Low
	Tozzi et al. 2004	Yes	Not clear	Yes	Yes	Yes	4	Low
	Vance et al. 2011	Yes	Not clear	Yes	Yes	Yes	4	Low
	Wagner et al. 2004	Yes	Yes	Yes	Yes	Yes	5	Low
	Waldrop-Valverde et al. (2006)	Yes	Not clear	Yes	Yes	Yes	4	Low
	Woods et al. 2008a	Yes	Not clear	Yes	Yes	Yes	4	Low
	Woods et al. 2017	Yes	Not clear	Yes	Not clear	Yes	3	Low
	Woods et al. 2008b	Yes	Not clear	Yes	Yes	Yes	4	Low
	Overall (average) RoB						4	Low

	Consistent?						Sign of effect size/effect range	Status
2	Impact of HAND on medication adherence						0.15–0.50	Yes
	Impact of HAND on activities of daily living						−0.0058 to 0.48	No
	Impacts of HAND on quality of life						0.037 to 0.46	Yes
	Impact of HAND on frailty						0.178 to 0.521	Yes

	Directness						No of studies with direct outcome	
3	Impact of HAND on medication adherence						12/13 (92%)	Yes
	Impact of HAND on activities of daily living						7/10 (70%)	Yes
	Impacts of HAND on quality of life						9/9 (100%)	Yes
	Impact of HAND on frailty						3/3 (100%)	Yes

	Precision						CI	
4	Impact of HAND on medication adherence						0.15–0.50	Yes
	Impact of HAND on activities of daily living						−0.0058 to 0.48	No
	Impacts of HAND on quality of life						0.037 to 0.46	No
	Impact of HAND on frailty						0.178 to 0.521	Yes

*RoB* Risk of bias; *CI* Confidence interval.

### Synthesis of results

All the studies included adult participants, with a mean age of 43.0 ± 6.3 years. Participants' age was similar for studies with different outcomes (medication adherence (45.3 ± 7.5 years), ADL (43.3 ± 2.5 years), QOL (37.9 ± 6.0 years) and frailty (49.9 ± 6.0 years). The overall ratio of men to women was 5:1. All studies had a similar distribution of male sex, irrespective of outcome measure (medication adherence (82.7 ± 7.7%), ADL (74.9 ± 27.8%) and QOL (72.4 ± 21.6%) and frailty (89.3 ± 9.7%)). Most participants were formally educated (≥ 7 years of formal education, with more than half having 13.3 ± 0.7 years of education. Mean years of education did not differ with outcome measures (medication adherence (13.1 ± 0.54yrs), ADL (13.7 ± 0.7yrs) and QOL (13.0 ± 1.0yrs)). Neuropsychiatric assessments were accounted for in about 75% of included studies. More than half (65%) of studies conducted comprehensive neuropsychological assessments. Only 18 (45%) studies had a control group, while the rest examined the relationship of an index of cognitive function with adherence, ADL, QOL and frailty. Cognitive function and impairment were measured using the Brief Inventory of Neurocognitive Impairment, International HIV dementia scale, MOS-cognitive functioning scale, A5001 Neuroscreen, Unified Parkinson's Disease Rating Scale, amongst others. Studies that employed comprehensive neuropsychological assessment used different definitions and criteria for diagnosing cognitive impairment in PLWHIV. Twenty (77%) studies used the global deficit score while 2 studies (7.7%) used the Frascati criteria to classify participants as either impaired or unimpaired. Only two studies^36,37^ investigated the association between cognition and more than one health outcome. Regarding confounding factors, 22 studies assessed substance abuse, 17 studies assessed alcohol use and 19 studies accounted for depression. Confounding factors were either included as exclusionary measures or as covariates, or both (Appendix 2).

**Figure 2:**
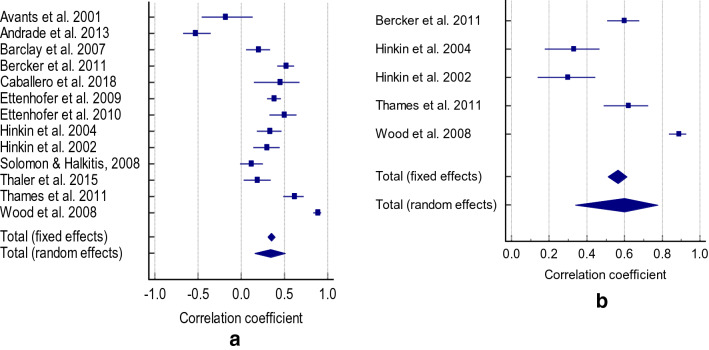
(**a**) Correlation between cognitive impairment and medication adherence (**b**) correlation between cognitive impairment and medication adherence.

Cognitive function/impairment was significantly associated with medication adherence (r = 0.339, CI 0.150 to 0.504, *p*  = 0.001; Z = 3.430, Q = 224.8897, I2 = 94.66%) (Fig. 2a). No publication bias was found (Eggers intercept =  −0.3999, *p* = 0.9219). When retaining studies that only assessed global cognitive impairment on medication adherence, the significant association was strengthened (r = 0.601, CI 0.338 to 0.776,  *p* = 0.001, Z = 3.980 Q = 80.1451, I^2^ = 95.01%) (Fig. 2b). There was no publication bias (Eggers intercept = 12.4203, p = 0.4041). The association between cognitive function and medication adherence was influenced by education (r = 0.79, *p* = 0.04) but not by age (r = 0.20, *p* = 0.505) or sex (r = 0.32,  *p* = 0.280).

**Figure 3:**
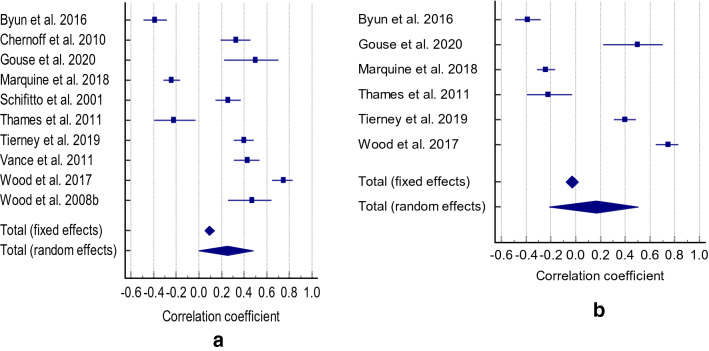
(**a**) Correlation between cognitive impairment and ADL (**b**) Correlation between cognitive impairment and ADL.

Cognitive functioning was not associated with ADL (r = 0.252, CI -0.0058 to 0.48, Z = 1.917, p = 0.055, Q = 324.14, I2 = 97.22%). No publication bias was found (Eggers intercept = 8.2756, *p* = 0.170) (Fig. 3a). After retaining only studies focusing on the impact of global cognitive impairment on ADL, the results remained non-significant (r = 0.167 (CI-0.215 to 0.505), Z = 0.853,  *p* = 0.393, Q = 244.7751, I^2^ = 97.96%) (Fig. 3b). Still, there was no publication bias (Eggers intercept = 7.3727, *p* =  0.3340). Only two studies examined the impact of cognitive function on employment, with effect sizes (r = 0.33)^38^ and (r = -0.26)^21^.

**Figure 4:**
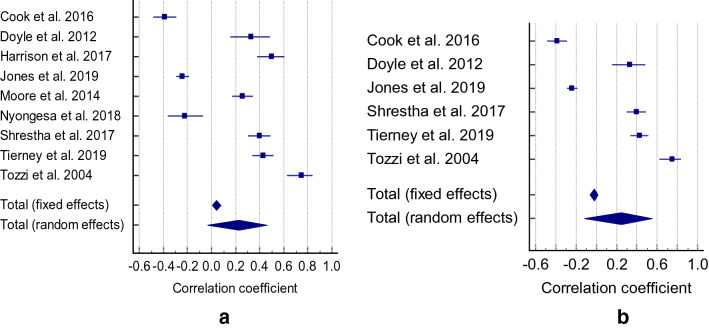
(**a**) Correlation between cognitive impairment and QOL (**b**) Correlation between cognitive impairment and QOL adherence.

Cognitive function was not associated with QOL (r = 0.227 CI 0.037 to 0.46), Z = 1.69,* p* = 0.091, Q = 418.8452, I2 = 98.09%), and there was no publication bias (10.15,* p* =  0.081) (Fig. 4a). The results did not change when including studies focusing on global cognitive impairment and QOL (r = 0.244 CI-0.117 to 0.548, Z = 1.334, P = 0.182, Q = 329.5619, I^2^ = 98.48% (Fig. 4b). Still, no publication bias was found (Eggers intercept = 11.6601, *p*= 0.1204).

**Figure 5:**
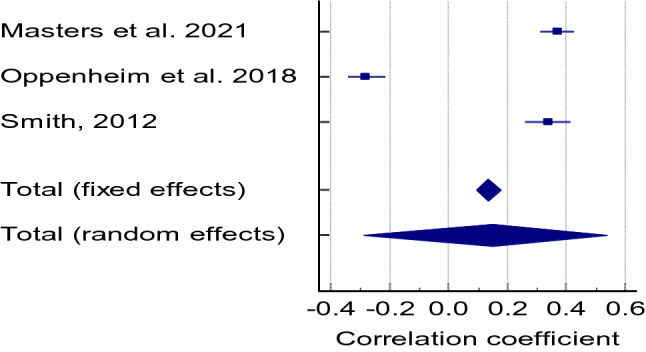
Correlation between cognitive function/impairment and frailty.

Cognitive function did not correlate with frailty (r = 0.196 CI = 0.178 to 0.521, Z = 1.027, * p* =  0.305, Q = 51.2884, I^2^ = 96.10%) (Fig. 5). We found only three studies that investigated the association between global cognitive impairment and frailty, of which two reported r values of 0.37^39^ and 0.34^40^.

## Discussion

### Summary of evidence

In this meta-analysis, we reviewed and synthesized existing evidence for the association between cognitive function and various outcomes in PLWHIV. Consistent with previous findings^41,42^, we found that declining global cognition is associated with lower medication adherence. Based on existing evidence, we did not find any associations between cognitive functioning and ADL, QOL or frailty. Numerous studies have assessed the functional consequences of cognitive impairment in PLWHIV, with most of the studies conducted in developed countries. Interestingly, we found that poor education was associated with poor cognitive function and adherence to medication, which has implications for the many PLWHIV in developing countries. Medication adherence was associated with poor cognitive function and lower education, but not with age or sex. According to Singh et al.^43^ and Tombaugh et al.^44^, lower education was associated with poor adherence and cognitive functioning. Although studies have shown that older age is associated with better adherence^42,45,46^, and worsening cognitive function^43,47,48^, we found that age did not influence the relationship between HIV-associated cognitive impairment and medication adherence. Similarly, female sex has been associated with better cognitive performance^43^, and better adherence^49^, but we found that sex did not influence the relationship between HIV-related cognitive impairment and medication adherence. Overall, our findings underscore the need to address cognitive functioning when designing interventions to improve medication adherence^50^. For example, PLWHIV who struggle with memory deficits could be encouraged to use compensatory strategies to improve organization and structure by using pillboxes, written times to take medications, digital reminders, among others^50^. Our findings also support repeatedly assessing cognitive functioning to detect individuals at risk of worsening adherence and deteriorating functional status^50^.

Although studies have shown that cognitive decline hurts ADL^51,52^, our review indicates inconsistent findings and reflects a small to moderate effect size in studies that reported significant associations between cognitive impairment and ADL in PLWHIV. Although HAND is common in the cART era, more than 90% of HAND cases represent asymptomatic and mild neurocognitive impairment subtypes^53^. In the modern era, HAND is thus unlikely to impact the ADL. Sanmarti et al.^54^ have also reported that cognitive impairment does not substantially interfere with ADL. Higher unemployment rates have been reported among PLWHIV, however, unemployment was not associated with cognitive impairment^55^. We found that cognitive function in PLWHIV was not associated with QOL, which contradicts a previous narrative review^3^. Alford and Vera^3^ employed narrative synthesis, unlike our synthesis which employed meta-analysis. Currently, the role of HAND in ageing is unclear, and some studies have suggested that HAND may lead to premature ageing^3^. Our meta-analysis suggests a weak relationship between cognitive functioning and frailty. This may be explained by most of the reviewed studies including relatively young (< 50 years) participants. Few studies have assessed interactions between HAND, age and frailty, leading to inconclusive findings.

People with HAND are unlikely to have poor QOL without remarkable interference in their ability to perform ADL. For hospitalized PLWHIV, the ability to return to performing ADL is an indication of good adherence and better QOL^56^. Our review involved mostly non-hospitalized PLWHIV and we found no evidence that cognitive-related declines in adherence led to cognitive-related declines in ADL or QOL. In the pre-cART era, HAND had various functional consequences including unemployment, poor medication adherence, and difficulty with driving and interpersonal functioning^24^. Our review suggests, that in the cART era, HAND does not bear significant functional consequences save for interfering with medication adherence, which in turn does not translate to poor ADL or QOL.

We observed a substantial degree of heterogeneity associated with the impact of HAND on medication adherence, which limits our interpretation of our findings. The observed heterogeneity may be due to differences in age, sex, education, occupation and setting. Hence, to solidify the inferences drawn from this review, further research is warranted to ascertain the role of occupation, age, sex and education in the perception of QOL and physical functioning in PLWHIV. Two individuals with the same level of impairment may perceive their QOL differently due to having different occupations. For example, bankers and customer care officers who have to multi-task may be more limited in their executive functioning compared to farmworkers. Currently, we require meta-analytic procedures that account for important sociodemographic factors such as age, sex, education and occupation in observational studies. Our findings are strengthened by the absence of publication bias. The cumulative confidence in this review seems high as most of the study possessed a low risk of bias, and evidence was consistent, direct and precise except for the impact of HAND on activities of daily living which were inconsistent and imprecise. Hence, further studies are needed to further explore the relationship of HAND and activities of daily living.

## Conclusions

Overall, in the modern cART era, HAND appears to have a significant impact on medication adherence, but little effect on ADL and QOL. Further studies are required to ascertain the impacts of HAND on frailty in the cART era, whilst accounting for age. Promoting medication adherence will have far-reaching effects for improving health outcomes for PLWHIV.

## Supplementary Information

Supplementary Information 1.

Supplementary Information 2.

## Data Availability

The datasets used and/or analysed during the current study are available from the corresponding author on reasonable request.
