# Author Correction: Impact of HIV-associated cognitive impairment on functional independence, frailty and quality of life in the modern era: a meta-analysis

**DOI:** 10.1038/s41598-022-14898-0

**Published:** 2022-06-20

**Authors:** Martins Nweke, Nombeko Mshunqane, Nalini Govender, Aderonke O. Akinpelu, Maryjane Ukwuoma

**Affiliations:** 1 Department of Physiotherapy, Faculty of Health Sciences, University of Pretoria, Pretoria, 0028 Hatfield South Africa; 2 Department of Basic Medical Sciences, Durban University of Technology, Durban, South Africa; 3 Department of Physiotherapy, University of Ibadan, Ibadan, Nigeria; 4 Department of Physiotherapy, University of Nigeria Teaching Hospital Ituku-Ozalla, Enugu, Nigeria

Correction to: *Scientific Reports* 10.1038/s41598-022-10474-8, published online 19 April 2022

The original version of this Article contained an error in the spelling of the author Nombeko Mshunqane, which was incorrectly given as Mshunqane Nombeko.

Additionally, in Table 1, part 2 column title,

“No of studies with indirect outcome”.

now reads:

“No of studies with direct outcome”.

The original Article has been corrected.

