# Supplementary material for: Impact of HIV-associated cognitive impairment on functional independence, frailty and quality of life in the modern era: a meta-analysis
Source: Sci Rep. 2022 Apr 19;12:6470. doi: 10.1038/s41598-022-10474-8 (PMC9019017; doi:10.1038/s41598-022-10474-8)
Supplement: Supplementary file 1 — Supplementary Information 1. [file 41598_2022_10474_MOESM1_ESM.docx]

**Appendix 1: Search strategies**

| Search terms | Database | Date of search | Filter | No. retrieved |
| --- | --- | --- | --- | --- |
| ((HIV OR human immune deficiency syndrome OR AIDS[MeSH Terms]) AND (cognition disorder[MeSH Terms]) AND (activities of daily living OR frailty OR quality of life OR sleep[MeSH Terms]) | PubMed | 1/02/2021 | Adults  1997-2021 | 575 |
| ((hiv or aids or acquired human immunodeficiency syndrome or human immunodeficiency virus (All Texts) AND (cognition disorder or cognition or cognitive function or cognitive performance or cognitive abilities or cognitive ability (All Texts) AND (medication adherence or medication compliance or medication nonadherence or medication noncompliance or frailty or frail or activities of daily living or adl or occupational performance or iadl or everyday functioning or daily functioning or quality of life or well-being or well-being or health-related quality of life or patient satisfaction or lived experience (All Texts)) | MEDLINE | 2/02/2021 | Adults  1997-2021 | 946 |
| ((hiv or aids or acquired human immunodeficiency syndrome or human immunodeficiency virus (subject Term) AND (cognition disorder or cognition or cognitive function or cognitive performance or cognitive abilities or cognitive ability (All Texts) AND (medication adherence or medication compliance or medication nonadherence or medication noncompliance or frailty or frail or activities of daily living or adl or occupational performance or iadl or everyday functioning or daily functioning or quality of life or well-being or well-being or health-related quality of life or patient satisfaction or lived experience (All Texts)) | Academic Search Complete | 2/02/2021 | Adults  1997-2021 | 1649 |
| ((hiv or aids or acquired human immunodeficiency syndrome or human immunodeficiency virus (All Texts) AND (cognition disorder or cognition or cognitive function or cognitive performance or cognitive abilities or cognitive ability (All Texts) AND (medication adherence or medication compliance or medication nonadherence or medication noncompliance or frailty or frail or activities of daily living or adl or occupational performance or iadl or everyday functioning or daily functioning or quality of life or well-being or well-being or health-related quality of life or patient satisfaction or lived experience (All Texts)) | CINAHL | 2/02/2021 | Adults  1997-2021 | 530 |
