# Supplementary material for: Impact of HIV-associated cognitive impairment on functional independence, frailty and quality of life in the modern era: a meta-analysis
Source: Sci Rep. 2022 Apr 19;12:6470. doi: 10.1038/s41598-022-10474-8 (PMC9019017; doi:10.1038/s41598-022-10474-8)
Supplement: Supplementary file 2 — Supplementary Information 2. [file 41598_2022_10474_MOESM2_ESM.docx]

|  | |  |  |  |  |  |  |
| --- | --- | --- | --- | --- | --- | --- | --- |
| Appendix 2: Search results for literature describing the association between HIV-associated neurocognitive disorder and adherence to medication, quality of life (QOL), activities of daily living and frailty. | | | | | | | |
| SN | Study ID Variables | Sample Size Sampling technique Study Design | Age X±SD (years) Sex (% Male) Education (Years) | Setting Country | Summary of findings | Outcome measure Confounds | Title of study |
| 1 | Ammassari et al. 2004 Cognition & adherence | n=135 Follow-up   Non-probability | Med age=35 Male: 64% ≤8yrs edu: 42% | Italy | Depressive symptoms but not HAND was associated with non-adherence. In bivariate analyses, poorer performance on Trails B was associated with non-adherence to ART. | Comp NP (Frascati criteria) Self-administered measure Alcohol: (-) Subst. abuse: (-) Neuromed + psych (+) Depression: (-) | Depressive symptoms, neurocognitive impairment, and adherence to highly active antiretroviral therapy among HIV-infected patients |
| 2 | Avants et al. 2001 Cognition & adherence | n=42 Cohort  Non-probability | Age=41.2 (5.2) Male: 69% 11.8 (2.1) | USA | Cognitive function was not associated with non-adherence (OR=-0.23 p=0.12, r=-0.18) | 4 NP tests Self-reported adherence.  Alcohol: (-) Subst. abuse: (-) Neuromed + psych: (-) Depression: (-) | Predictors of non-adherence to HIV-related medication regimens during methadone stabilization. |
| 3 | Andrade et al. 2013 Cognition & adherence | n=80 Prospective follow-up  Non-probability | Age: 45(±7.5) Male: 79% 12.6±2.2 | Medical Centre  USA | Mean adherence was 86.4%. After replacing GDS (−4.0; p=0.083), with each of the 7 NP domains, only one, working memory, significantly affected adherence ((OR (SE) =−4.9 (2.2); p=0.026, r= -0.53) | Pharmacy refill adherence method Comp NP (GDS)  Alcohol (40%) Subst. use (83%) Depression (49%) ART experience: median of 8months. Median duration of HIV: 127 (73–179) Neuromed: + Psych exam: + | Relationships among neurocognitive status, medication adherence measured by pharmacy refill records, and virologic suppression in HIV- infected persons |
| 4 | Barclay et al. 2007 Cognition & adherence | n=185 Cross-sectional  Convenience | Age: 44(7.3) Male: 78% Edu: 13.1(2.3) | Medical Centers  USA | Older participants who did not adhere to HAART performed poorly on measures of learning and memory, executive functioning, and global functioning. Poor adherence was associated with weaker performance on measures of learning and memory (t(43) = 2.07, p =0.05, R=0.2) and executive functioning (t(43) = 2.59, p = .01, R=0.2). | Comp NP MEMS cap  Alcohol: (10.3%) (-) Subst. use: 9.7% (-) Depression: (+) Neuromed: (+) Psyc: (+) | Age-associated predictors of medication adherence in HIV-positive adults: Health beliefs, self-efficacy, and neurocognitive status |
| 5 | Becker et al. 2011 Cognition & adherence | n=215 Longitudinal  Non-probability | Age: 41.6 (7.3) Male: 176 (82%) Edu: 13±2.15 | Infectious disease clinics Community  USA | Global deterioration scale (GDS) associated with adherence (OR=6.3, r=0.60). Learning and memory associated with changes in adherence (OR=3.44, r=0.43). | MEMS caps Comp NP (GDS)  Alcohol: (-) Subst. use: 69% (-) Depression: (-) ART experience: 6.5 yrs Duration of HIV: not reported. Neuromed: (+) Psych: (+) | Longitudinal change in cognitive function and medication adherence in HIV-Infected Adults |
| 6 | Caballero et al. 2018 Cognition & adherence | n=37 Prospective follow-up Non-probability | Age: 68.8 (3.2) Male: >90% Edu: ≥high school: 97% | Dental clinic  USA | CLOX 1 and GPB significantly related to adherence (P < 0.05) (R = 0.514 vs R = 0.381). | CLOX 1 for executive function  Grooved Peg board (GBP) for motor skills MEMS cap Alcohol: (-) Subst. use: (-) Neuromed + psych: (+) Depression: (-) | Association between cognitive tests and antiretroviral medication adherence in older adults with HIV |
| 7 | Ettenhofer et al. 2009 Cognition & adherence | n=431 Cross sectional  Convenience | Age: 42.79 (7.22) Male: 80.5% Edu: 13.02±2.19 | Community agencies & medical center  USA | Young age: Cognition not associated with adherence. Older age: Cognition significantly related to both MEMS adherence (r = 0.38, z = 2.85, p = 0.004) and qualitative self-report of adherence (r = 0.31, z = 2.40, p=0.02) | Comp NP (GDS) MEMS Self-report of adherence  Alcohol: (+) Subst. abuse: (+) Neuromed + psych (+) Depression: (+) | Aging, neurocognition, and medication adherence in HIV Infection |
| 8 | Ettenhofer et al. 2010 Cognition & adherence | n=91 Prospective cohort  Convenience | Age: 42.25 (7.7) Male: 71 (78%) Edu: 13.23 (2.22) | Community agencies & medical center  USA | Better global cognition predicted better medication adherence over six months (OR=0.23, r=0.5) p*=*0.03) | Comp NP t-score MEMS caps  Alcohol: (+) Subst. abuse: (+) Neuromed + psych (+) Depression: NAF (-) Other comorbidities: exc. | Reciprocal prediction of medication adherence and neuro-cognition in HIV/AIDS |
| 9 | Hinkin et al. 2004 Cognition & adherence | n=148 Cross-sectional  Convenience | Age: 44.2 (7.7) Male: 83% Edu: 13.4 (2.3) | Medical centers and HIV community agencies  USA | Neurocognitive impairment conferred a 2.5 times greater risk of poor adherence (OR 2.5, 95% CI 1.19–5.35, r=-0.33). | Comp NP (GDS) MEMS caps  Alcohol: (+) Subst. use: (+) Psych: (+) Neuromed: (-) Depression: (+) | Medication adherence in HIV-infected adults: effect of patient age, cognitive status, and substance abuse |
| 10 | Solomon and Halkitis 2008 Cognition & adherence | n=213 Follow-up  Non-probability | Age: 42 Male: 100% Edu:- | Bars, cafes, dance clubs, sex clubs, and on streets   USA | At baseline, HAART adherent participants evidenced less executive functioning impairment than non-adherent participants. This association did not reach statistical significance in 10-month follow-up (r=0.12) | Executive functioning measured by Trail making Test A and B. Adherence by MEMS cap  Alcohol: (-) Neuro + psych: (-) Substance use: (-) Depression : (-) | Cognitive executive functioning in relation to HIV medication adherence among gay, bisexual, and other men who have sex with men. |
| 11 | Hinkin et al. 2002 Cognition & adherence | n=137 Cross-sectional  Probability | Age: 44.06 (7.53) Male: 82% Edu: 13.42 (2.34) | Medical centers and HIV community agencies  USA | Mean adherence was 80.2% (SD 21.0). Neuropsychological compromise was associated with a 2.3 (95% CI 1.01-5.51, r=0.30) times greater risk of adherence failure. | Comp NP (GDS) MEMS cap  Alcohol: (+) Subst. abuse: (+) Psych: (+) Neuromed: (+) Depression: (-) | Medication adherence among HIV+ adults: Effects of cognitive dysfunction and regimen complexity |
| 12 | Levine et al. 2005  Cognition & Adherence | n=222  Follow-up  Non-probability | Age: 44 Male: 80% Edu: 13.1 | USA | Worst performers on the global impairment index and attention and working memory domain: Poor weekend adherers | Comprehensive NP MEMS cap Alcohol: (-) Subst. abuse: (-) Psych: NAF (-) Neuromed: (-) Depression: (-) | Variations in patterns of highly active antiretroviral therapy (HAART) adherence |
| 13 | Thaler et al. 2015 Cognition & adherence | n=150 Cohort Non-probability | Age: 41.9 (7.4) Male: 82% Edu: 13.1 (2.1) | HIV clinics   USA | Increases in mean neurocognition positively correlated with adherence (r = 0.19, *p* = 0.019). | Comp NP (T-score) MEMS cap  Alcohol: (-) Neuro + psych: (+) Substance use: (+) Depression: (+) | Increased neurocognitive intra-individual variability is associated with declines in medication adherence in HIV infected adults |
| 14 | Wagner (2002) Cognition & adherence | n=180 Follow-up  Non-probability | Age: 41 Male:82 Edu: NR | USA | Lower adherence associated with  worse performance on CVLT and Trails A when using EM rates of adherence and lower scores on Digit Symbol when using MD and SR adherence rates | Self-report, medication diaries, and electronic monitoring were used to assess adherence  Alcohol: not clear Substance use: not clear Neuro +psych: not clear Depression: not clear | Predictors of antiretroviral adherence as measured by self-report, electronic monitoring, and medication diaries. |
| 15 | Wagner et al. (2004) Cognition & adherence | n=45 Follow up  Non-probability | Age: 42 Male:79 Edu: NR | USA | Mean adherence rate was 66% and not associated with neuropsychological variables. | Trail making Test A and B Digit Symbol, WAIS-R. MEMS cap | Correlates of HIV antiretroviral adherence in persons with serious mental illness. |
| 16 | Waldrop-Valverde et al. 2006 Cognition & adherence | n=57 Cross-sectional   Non-probability | Age: 42 Male: 77% Edu: NR | USA | Only psychomotor slowing was significantly associated with non-adherence. | Comp NP Self-report of adherence  Alcohol: NE (+) Substance use: NE (+) Neuro + psych: Exc (+) Depression: NE (+) | Neurocognitive aspects of medication adherence in HIV-positive injecting drug users |
| 17 | Woods et al 2008a Cognition & medication management | n=87 Cross-sectional  Non-probability | Age: 45.9 (8.8) Male: 89.7% Edu: 13.8 (2.8) | HIV clinics Community  USA | Medication management & NCI were positively associated (R=0.89, p=0.013). | Comp NP (T-score) The Prospective Memory for Medications Questionnaire  Alcohol: (+) Substance use: (+) Neuro + psych: (+) Depression: (+) | Prospective memory in HIV infection: Is “Remembering to Remember” a Unique Predictor of Self-reported Medication Management |
| Relationship of Cognitive Function/impairment with ADL among PLWHIV | | | | | | | |
| 18 | Byun et al. 2016 Cognition & Sleep disturbance | n=268 Cross-sectional   Non-probability | Age: 44.8 (8.5) Male: 179 (67%) Edu: 10010>high school (56%) | Hospital, community  USA | Sleep disturbance associated with cognitive impairment (R=−0.39) | Pittsburgh Sleep Quality Index Medical Outcome Study (MOS) Cognitive Functioning Scale  Alcohol: (-) Subst use: (+) Neuromed + psyc: (+) Insomnia: (-) Depression: (-) | Sleep, fatigue and problems with cognitive function in adults living with HIV |
| 19 | Chernoff et al. 2010 Cognition & employment | n=174 Retrospective from a randomized controlled trial   Probability | Age: 44.1 (7.7) Male: 158 (90.8%) Edu: 14.9±2.8 | Community  USA | Executive functioning weakly predicted employment status (χ2= 19.42, *p* = 0.002; R= 0.33 (CI 0.19-0.50) | Comp NP (GDS) Self-reported measure of employment activities Alcohol: (+) Subst. use: (+) Neuromed + psych (+) Depression: (+) | Neuropsychological functioning as a predictor of employment activity in a longitudinal study of HIV-infected adults contemplating workforce reentry |
| 20 | Gouse et al. 2020 Cognition & ADL (driving) | n=40 Follow-design  Non-probability | Age: 39.2 (7.05) Male: 100% Edu: 50% ≥12 yrs | Health care clinics and institutions & social media outlets  South Africa | Professional drivers with NCI have a higher risk of making driving errors under high-risk conditions compared to their neurocognitive normal counterparts (U=73.50, r= 0.50) | Cognition: Comp NP (GDS) Driving: 2 interactive driving simulations The self-report Driving History and Habits (DHH)  Alcohol: (+) Subst. use: (+) Neuro + psych: (+) Depression: (-) | The impact of HIV‑Associated neurocognitive impairment on driving performance in commercial truck drivers |
| 21 | Marquine et al. 2018 Cognition & activities of daily living & unemployment | n=670 Cohort  Non-probability | Age: 42.7 (10.0) Male: 88.36% Edu: 13.5 (2.5) | Community  USA | NCI and functional outcomes (IADL) were significantly associated (p<0.01, OR (SE)=0.75 (0.19), r=-0.11, PAOFI-OR (SE)= 2.75 (0.58), r=-0.36) & unemployment (OR (SE): 2.04 (1.40–3.00), r=-0.26) | Comp NP (GDS) Lawton Scale & PAOFI1  Alcohol: (+) Subst. use: (+) Psych: (+) Neuromed: (+) Depression: (+) | A composite of multisystem injury and neurocognitive impairment in HIV infection: association with everyday functioning |
| 22 | Schifitto et al. 2001 Cognition & physical function | n=270 Cohort  Non-probability | Age: 39.8 (7.6) Male: 79.4% Edu: 13.6 (3.1) | Hospital  USA | Correlation between physical function and the Psychomotor Speed (not executive function & attention/memory) *was significant r=*0.26, *p=*0.003). | Rey Auditory Verbal Learning Test Psychomotor Speed Grooved Pegboard Symbol Digit Modalities Test Rey Complex Figure Copy Immediate Recall Odd-Man-Out Test Ve23r31bal Fluency Karnofsky Performance Scale for physical function  Alcohol: not clear Subst. use: not clear Psych: done: not clear Neuromed: not clear Depression: not clear | Clinical trials in HIV associated cognitive impairment: Cognitive and functional outcomes |
| 23 | Thames et al. 2011 Cognition & ADL driving & medication management | n=107 Cross-sectional  Non-probability | Age: 49.1 (11.1) Male: 81% Edu: 13.7 (2.2) | Medical centers Community agencies  USA | Neuropsychological functioning associated with medication management (r = 0.617, p < 0.001) and errors produced on the driving simulator (r = −0.219, p =0.024) | Comp NP (GDS) Self-report of cognitive function Columbia Medication Management Task – Revised Laboratory-based driving simulator task (STISIM driving version 2.0)  Alcohol: (+) Neuro + psych: (+) Substance use: (+) Depression : (+) | Depression, cognition and self-appraisal of functional abilities in HIV: An examination of subjective appraisal vs. objective performance |
| 24 | Tozzi et al. 2004 Cognition & QoL | n=70 Cross-sectional  Non-probability | Age: 36.1 (23-57) Male: 70% Edu: 11.6 (5-18) | National institute for infectious diseases Italy | QOL was significantly different for unimpaired:45.5 (23.1) and impaired: 37.0 (21.1). | Comp NP QOL by MOS-HIV health Survey  Alcohol: (+) Neuro + psych: (+) Substance use: (+) Depression: (-) | Neurocognitive impairment influences quality of life in HIV-infected patients receiving HAART |
| 25 | Vance et al. 2011 Cognition & ADL | n=201 Cross-sectional  Non-probability | Age: 45.25 (23 – 67) Male: 71.4% Edu: NA | HIV clinics & community  USA | Poorer cognitive performance associated with poorer performance on the Timed Instrumental Activities of Daily Living (r=0.43, p<0.05). | Comp NP  Timed instrumental activities of daily living scale  Alcohol: (-) Neuro + psych: (+) Substance use: (+) Depression : (+) | Cognitive and everyday functioning in older and younger adults with and without HIV |
| 26 | Woods et al 2017 Cognition & ADL | n=93 Cross-sectional  Non-probability | Age: 45.2 (10.5) Male: 11.6% Edu: 13.1 (2.1) | HIV clinics Community  USA | HAND associated with a higher failure rate on S-MarT (p <0.05, OR: 13.6 (CI=1.6–110, r=0.75) as compared to the HIV− and HAND− groups, respectively. | Comp NP (GDS) Lawton scale Karnofsky Scale of Performance  Alcohol: (+) Substance use: (+) Neuro + psych: (+) Depression: (+) | Household everyday functioning in the internet age: Online shopping and banking skills are affected in HIV-associated neurocognitive disorders |
| 27 | Woods et al. 2008b Cognition & ADL | n=66 Cohort  Non-probability | Age: 45.0 (8.3) Male: 90.9% Edu: 13.4 (2.9) | HIV clinics Community  USA | Prospective memory was associated with ADL (r=0.47). | Comp NP (GDS) Lawton and Brody ADL scale  Alcohol: Not sure Subst. use (53%) (-) Neuro + psych: (+) Depression (48.5%) (-) | HIV-associated prospective memory impairment increases risk of dependence in everyday functioning |
| 28 | Tierney et al. 2019 Cognition & ADL + QOL | n=336 Longitudinal  Non-probability | Age: 28.2 (6) Male: 67.1% Edu: 13.8 (2.3) | HIV clinics Community USA | Older HIV+ adults: mild extrapyramidal symptoms increase the risk of daily functioning problems (β = 3.08, p = 0.003, r=0.40) and lower health-related physical QoL (β= −2.55, p=.01, r=-0.34). | Comp NP (UPDRS)  Lawton & Broody IADL scale MOS SF-36  Alcohol: (-) Neuro + psych: (+) Substance use: (+) Depression: (+) | Extrapyramidal motor signs in older adults with HIV disease: frequency, 1-year course, and associations with activities of daily living and quality of life |
| Relationship of Cognitive Function/impairment with Quality of Life among PLWHIV | | | | | | | |
| 29 | Cook et al. 2016  Cognition & QOL | n=302 Cross-sectional  Non-probability | Age: 29±3yrs Male: 63% Edu: 62% | Hospital & community  Northern India | Cognitive impairment & quality of life were not associated ((OR (SE) = −0.49 (1.02); p=0.632, r=0.13) | IHDS WHO-QOL bref  Alcohol: not assess (-) Subst. use: (-) Neuromed + psych (-) Depression: (-) | HIV Clade-C infection and cognitive impairment, fatigue, depression, and quality of life in Early-Stage Infection in Northern Indians |
| 30 | Doyle et al. 2012 Cognition & QOL | n=113 Cohort  Non-probability | Age: 43.4±5.4 Male: 12.9±2.3 Edu: 82.4% | Hospital & community  USA | HAND and mental HRQOL were associated (OR=0.11; p<0.0, r=-0.68) but not physical HRQoL ( OR=2.44; P=0.19) | Comp NP (GDS) RAND 36-Item Short Form Health Survey (SF-36)  Alcohol: (+) Subst. use: (+) Neuromed + psych (+) Depression: (+) | Aging, prospective memory, and health-related quality of life in HIV infection |
| 31 | Harrison et al. 2017 Cognition & QOL | n=173 Cross-sectional  Probability | Age: 42.7(11) Male: 64% Edu: ≥high school: 97% | Media adverts & clinical sites  USA | Higher discrimination index on the N-back was associated with better HAT-QoL Health (OR=3.6, p=0.02, r=0.45). | The Penn Continuous Performance Task & N-back task QOL: The HIV/AIDS-Targeted QoL (HAT-QoL)  Alcohol: (+) Subst. use: (+) Neuro + psych: (+) Depression: (+) | The nature and consequences of cognitive deficits among tobacco smokers with HIV: A comparison to tobacco smokers without HIV |
| 32 | Jones et al. 2019 Cognition & QoL | n=1306 Cohort  Non-probability | Age: 42.0 (8.8) Male: 100% | Academic Institutions  USA | Global cognition was associated with physical Qol (OR=0.040; P<0.01, r=0.80) & mental QoL (OR=0.016, r=0.91). | Cognition: Comp NP HRQoL: SF-36  Alcohol: not clear Subst. use: (-) Psych: (+) Neuromed: (+) Depression: (+) | Changes in cognition precede changes in HRQoL among HIV+ males: Longitudinal analysis of the multicenter AIDS cohort study |
| 33 | Mayo et al. 2020 Cognition & QOL | n=707 Cross-sectional  Non-probability | Age: 53.4 (8.3) Male: 100% Edu: 14.0 (2.5) | HIV clinics Canada | Cognitive performance is an important contributor to QOL. | Cognitive ability: B-CAM WHOQoL  Alcohol: (_-_) Subst. use: (+) Psych: (+) Neuromed: (+) Depression: not sure | Relationships between cognition, function, and quality of life among HIV+ Canadian men |
| 34 | Moore et al. 2014 Cognition & HRQoL | n=302 Cross-sectional Non-probability | Age: 44.1 (4.7) Men: 76.4% Edu: 13.8 (2.5) | Community org Substance use recovery clinics, HIV community organizations, and HIV treatment clinics  USA | Successful cognitive aging predicted Mental HRQoL (β = 0.41, *p* < 0.01, r=0.3) but not Physical HRQoL (β = 0.21, *p =* 0.12, r=0.18). | Comp NP MOS-SF-36  Alcohol: (_+_) Subst. use: (+) Psych: (+) Neuromed: (+) Depression: not sure | Successful cognitive aging and health-related quality of life in younger and older adults infected with HIV |
| 35 | Nyongesa et al. 2018 Cognition & QoL | n=167 Cross-sectional Mixed ST | Age: 37.0 (8.7) Male: 28.7% Edu: ≥primary 39.4% | HIV clinic & Community Kenya | No correlation between neurocognitive scores and QOL (r=−0.05, p=0.63). | NeuroScreen, Swahili version RAND SF-36.  Alcohol: (-) Subst. abuse: (-) Psych: not clear Neuromed: not clear Depression: not clear | Neurocognitive and mental health outcomes and association with quality of life among adults living with HIV: a cross-sectional focus on a low- literacy population from coastal Kenya |
| 36 | Shrestha et al. 2017 Cognition & HRQoL | n=311 Cross-sectional  Probability | Age: 38.9 (6.8) Male: 100% Edu: >primary 80.6% | Prisons  Malaysia | Significant association between NCI & HRQOL (B = −.302, p<0.05, r=-0.42) | Brief Inventory of Neurocognitive Impairment (BINI)  RAND 36-Item Health Survey (SF-36) for HRQOL  Alcohol: (-) Subst. use: not sure Psych: (+) Neuromed: not sure Depression: (+) | The influence of neurocognitive impairment, depression, and alcohol use disorders on health-related quality of life among incarcerated, HIV-infected, opioid dependent Malaysian men: A moderated mediation analysis |
| Relationship of Cognitive Function/impairment with Frailty among PLWHIV | | | | | | | |
| 37 | Erlandson et al. 2018 Cognition & Frailty | n=987 Prospective follow-up  Probability | Age: 54 (50–57) Male:81%- | Hospitals & clinical research centres  USA | Frailty was associated with NCI with more than double the risk of poor health outcomes (PR 2.65; 95% CI 1.98, 3.54). NCI was associated with poor health outcomes (PR 1.73; 95% CI 1.36, 2.20). | A5001 Neuroscreen (Trail Making A, Trail Making B, and Digit Symbol tests) IADL-Lawton-Brody scale Self-report of falls  Alcohol: (+) Subst. use: (+) Neuromed: (+) Psych: (+) Depression: (+) | Frailty, neurocognitive impairment, or both in predicting poor health outcomes among adults living with Human Immunodeficiency Virus |
| 38 | Masters et al. 2021 Cognition & frailty | n=929 Cohort  Probability | Age: 51 (7.41) Male: 81% Edu: ≥high school: 95. 4% | USA | NCI and frailty were strongly associated (aOR=2.79; 95% CI=1.21, 6.43; p=0.02, r=0.37 | A5001 Neuroscreen Fried Frailty phenotype with grip strength 4-meter walk speed Self-reported low activity, exhaustion, and unintentional weight loss   Alcohol: (_+_) Subst. use: (+) Psych: (+) Neuromed: (+) Depression: (+) | Baseline neurocognitive impairment (NCI) is associated with incident frailty but baseline frailty does not predict incident NCI in older persons with HIV |
| 39 | Oppenheim et al. 2018 Cognition & frailty | n=811 Cross-sectional  Non-probability | Age: 44.6 (11.2) Male: 86.9% Edu: 13.4 (2.6) | Clinical Research Centre  USA | Global NP mean SS was negatively associated with the frailty index (r = −0.28, p < 0.001), indicating increased frailty with worsening global cognitive ability. | Comp NP (GDS) The frailty index was constructed using 26 factors.  Alcohol: (-) Subst. use: (-) Psych: (+) Neuromed: (+) Depression (14.6%) (-) | Neurocognitive functioning predicts frailty index in HIV |
| 40 | Smith 2012 Cognition & frailty | n=505 Cohort  Non-probability | Age: 52 (median) Male: 100% Edu: >12yrs of =84% | USA | Participants with HAND had a 2.18-2.99 odds ratio for the frailty phenotype (CI=1.05; P=0.036, r=0.34) | Comp NP (Frascati)  Alcohol: NR Subst. use (31%) (-) Psych: not sure Neuromed: not sure Depression (21.6%) (-) | Association of HIV-associated neurocognitive disorder with frailty in hiv-1 seropositive men |

(-): not accounted for; (+): accounted for; Comp NP: comprehensive neuropsychological; GDS: global deficit score
